# Radiation therapy and serum salivary amylase in head and neck cancer

**DOI:** 10.18632/oncotarget.18763

**Published:** 2017-06-28

**Authors:** Francesca De Felice, Mario Tombolini, Angela Musella, Francesco Marampon, Vincenzo Tombolini, Daniela Musio

**Affiliations:** ^1^ Department of Radiotherapy, Policlinico Umberto I, “Sapienza” University of Rome, Rome, Italy; ^2^ Department Organs of Sense, Policlinico Umberto I, “Sapienza”, University of Rome, Rome, Italy; ^3^ Department of Gynecology, Obstetrics and Urological Sciences, Policlinico Umberto I, “Sapienza” University of Rome, Rome, Italy; ^4^ Department of Biotechnological and Applied Clinical Sciences, Laboratory of Radiobiology, University of L'Aquila, L'Aquila, Italy

**Keywords:** amylase, head and neck cancer, radiotherapy, dose, salivary glands

## Abstract

Radiation therapy (RT) is a valid treatment option for head and neck cancer (HNC). The risk of RT-induced toxicities is significant, especially due to extended treatment fields. The raise in amylase activity is strictly dependent on the volume of salivary glands included in the irradiated target volume and it is firmly related to the dose. The aim of this review is to report the effects on salivary amylase activity after radiation exposure of salivary glands, in patients with HNC.

## INTRODUCTION

Radiotherapy (RT) represents one of the classical options for managing head and neck cancer (HNC). Radiation effects on salivary glands are of particular interest in clinical practice. Generally, salivary glands, especially parotid glands, show a high sensitivity to ionizing radiations, although they have a very low proliferative activity [1]. The increase in serum salivary amylase secondary to salivary glands irradiation is of considerable radiobiological and radiotherapeutic significance. This phenomenon has been mainly ascribed to the disruption of serous cells and changes in their cell membrane permeability resulting in intracellular amylase release. Ionizing radiations cause loss of acinar architecture, inflammatory cell infiltration and disruption or vacuolation of serous cells [2].

The purpose of this narrative review is to provide an updated description of early irradiation effects on salivary glands in HNC. We focus on variations of serum salivary amylase after RT. We will briefly introduce the salivary amylase property in an attempt to provide a means of understanding how it relates specifically to irradiation. We give a summary of clinical research focused on evaluating changes in serum amylase levels. It represents a distinctive opportunity for learning more about the clinical radiobiology of salivary amylase during and after RT in HNC patients.

### Basic knowledge of salivary amylase

Saliva plays a central role in dental and oral care, in lubricating mucosa, in contributing to antimicrobial activity and facilitating eating. Submandibular glands produce about 70% of saliva, whereas 20% of saliva is secreted by parotid glands and approximately 10% by minor salivary glands [3]. Saliva has a crucial roles in food boli preparation and digestion [4]. Saliva contains several proteins involved in digestive process and salivary amylase is the most abundant components. Salivary amylase is mainly produced in the parotid gland and it is responsible for starch hydrolysis, initiating carbohydrate digestion in the oral cavity. It is a calcium-containing metallo-enzyme that hydrolyzes the α-(l,4)-linkages of starch to glucose and maltose [5]. The molecular weight is about 55,000 and the whole saliva may contain 0.04 to 0.4 mg/ml amylase [6]. Salivary amylase level varies during the day, from individual to individual, depends on different regions in the oral cavity and tends to diminish with old age. Salivary amylase concentration can be detected in the blood. The reference range for serum amylase is 40–140 U/L. But serum amylase is produced by both salivary glands and exocrine pancreas at almost equal proportion [4]. The most accurate method for distinguishing salivary amylase from pancreatic amylase is the isoamylase analysis by agarose gel electrophoresis [7]. On agarose gel electrophoresis, the mobility of the more anionic isoenzyme is related to salivary amylase, whereas the less anionic band corresponds to pancreatic amylase. Physiologically, based on carbohydrate content, a total of five isoenzymes are determined, three isoenzymes of salivary amylase (which contain carbohydrates) and two isoenzymes of pancreatic amylase (which no contain carbohydrates) [8].

According to a classical view, elevated concentration of digestive enzymes in the serum, including amylase and lipase, can suggest an inflammatory diseases such as acute pancreatitis. Whereas elevation in serum amylase levels with no increase in serum lipase concentration or apparent pancreatic disorder can be used in the diagnosis of salivary glands inflammation or acute stress.

### Salivary amylase: the radiobiological properties

Post RT hyperamylasemia is almost only related to salivary glands irradiation [9]. Although irradiation-induced damage in salivary gland was first described in 1911, its mechanism is still a de- bate [10]. Salivary glands tissue is largely differentiated and metabolically active with a low mitotic rate and no expected future mitoses [11]. Thus, based on Bergonie and Tribondeau radiosensitivity law, salivary glands are presumably radioresistant. Radiation-effect on mechanisms of salivary gland radiosensitivity has been tested mainly in parotid glands, whereas submandibular, sublingual and minor salivary glands have been studied lesser in term of response to irradiation. Based on experiments with rodents and especially with rats, the regional differences in their parotid gland radiosensitivity have been considered as plausible model for human circumstances [12]. The cranial part of the parotid gland is more radiosensitive than the caudal part. Ionizing radiation induces damage to muscarinic receptors involved in secretary responsiveness, with consequent destruction of serous cells [13]. Secretory granules of serous cells are rich in proteolytic enzymes. This characteristic can justify the high radiosensitivity of the parotid glands, which contain much higher level of mucous cells, compared with the relative radioresistance of the other salivary glands [3].

**Figure 1:**
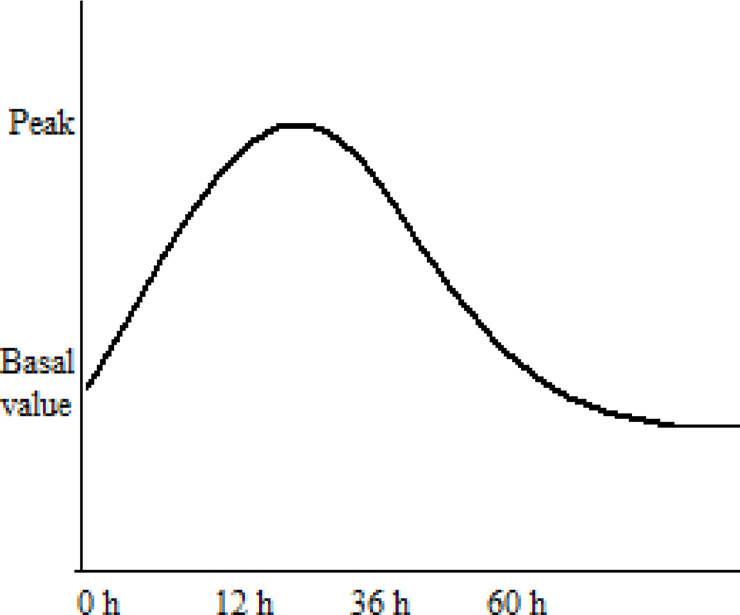
Qualitative representation of salivary amylase kinetics after irradiation

The time kinetics of damage is expressed in two phases: at first (0–60 days) salivary gland dysfunction depends on plasma membrane damage, later (60–240 days) on “traditional” killing of progenitor cells [14]. Figure 1 summarizes salivary amylase kinetics after irradiation, using a qualitative curve. Irradiation of the salivary glands determines a rapid increase in the serum amylase within a few hours after irradiation and a return to normal levels in the subsequent days. During the acute phase of radiation damage, within few hours after dose up to 4 Gy, the amylase secretion is quickly affected, with a 10 to 80 fold increase of the serum value. This phenomenon reaches its peak within 12–36 hours and it seems to be a direct consequence of acinar cells impairment [11]. Later on the amylase production deteriorates, due to newly formed acinar cells that are unable to work properly. Therefore, hyperamylasemia represents a very early change in serous salivary cells, that loss their ability to produced amylase.

### Radiation therapy and salivary amylase: the clinical trials

Few studies are available in literature investigating early radiation-induced amylase changes in HNC patients (Table 1). Globally, they described some correlation between the degree of amylase rise found in the serum and the amount of salivary tissue included in the treatment volume. The increase in enzyme activity, as well as its peak value, depends on the fractionation modality. It is higher and appears earlier with an increasing daily delivered total dose. The vast majority of data involved parotid glands, due to their well-documented amylase activity, instead of submandibular, sublingual and minor salivary glands.

**Table 1:** Clinical head and neck studies on serum amylase increase

Author	Patient population (*n*)	Salivary amylase peak (after first RT fraction)
Leslie [2]	head and neck cancer (41)	24–48 hours
Kashima [15]	head and neck cancer (33)	9–36 hours
Borok [16]	head and neck cancer (12)	48–96 hours

RT: radiotherapy.

Leslie et al. measured serum amylases prior and at 24-h intervals following the start of RT in patients with locally advanced HNC [2]. Treatment plan included opposed fields in order to cover the whole neck up to above the hard palate. Thus all the major salivary glands were included in the treatment volume. Two different fractionation schedules were used, including continuous hyperfractionated accelerated RT and conventional fractionated RT. Marked rises in the serum amylase were seen in all these patients peaking 24–48 hours after the irradiation and then falls rapidly reaching normal levels. The peak rise in serum amylase was greater in hyperfractionated accelerated schedule than in conventional fractionated RT (mean peak 4477 IU/l and 3275 IU/l, respectively). Similar results were obtained by Kashima et al. [15] and Borok et al. [16]. Kashima et al. analyzed 33 patients with HNC cancer and noted a transient hyperamylasemia after a dose range of 1–2.75 Gy, with a peak elevation at 9–36 hours after RT [15]. Borok et al. measured serum amylase levels in 12 consecutive HNC patients treated with fractionated doses from 1.8 to 4 Gy per day in accordance with patient's needs [16]. Rapid transient increases in enzyme activity, limited to the first days of RT, were consistently found. Interestingly, van den Brenk et al. [17] studied the effects of steroids administration on post-RT changes in serum amylase, in order to protect salivary tissues from radiation damage. Results were obtained in 12 patients with HNC receiving 100 or 200 mg prednisolone intravenously approximately 20 minutes before the first RT fraction. Prednisolone reduced the post-RT hyperamylasaemic response compared with that following the second fraction in which prednisolone was not administered before treatment. Perhaps, the steroid decreased damage to salivary tissue, by influencing the labilisation and stabilisation of lysosomal membranes. However, little evidence has been provided to support the hypothesis that prednisolone should be administered in a prophylactic way.

TBI represents a situation not generally encountered in HNC clinical practice. In fact, HNC patients are treated with RT fractionated doses: a conventional fractionation of 2 Gy per day, five times a week, or a hypofractionated scheme with a simultaneously integrated boost (SIB) technique. The parotid glands received at least 2 Gy per day. However, HNC treatment that includes salivary gland in the target volume, had a similar rise in amylasemia as did TBI patients who had received the same dose. Published data concerning acute effects after TBI are also consistent with dose-response relationship. Junglee et al. [18] tested whether TBI may cause different changes in pancreatic and salivary amylase serum concentration. Six leukemic patients received TBI (total dose 7.5 Gy) before bone marrow transplantation. Biochemical results demonstrated that salivary amylase activity increased by 21 to 51 fold the basal value, peaking on the first day after TBI and returning to normal within five days. Whereas the pancreatic enzymes after irradiation showed minimal or no change. The increase of salivary amylase was associated with the development of clinical parotitis. All patients reported swelling in parotid and submandibular glands region immediately after TBI and it subsided over 24–36 hours. Analogous results were previously defined by Barrett et al. [19]. Most of the serum amylase increase derived from salivary damage, with a much smaller pancreatic component. This rise was associated with symptoms of acute parotitis in all 12 acute leukaemia patients undergoing TBI (total dose 10 Gy) for bone marrow trasplantation. At Institut Gustave-Roussy, Hennequin et al. studied amylasemia in 38 patients [9]. Of these patients, 15 patients received TBI for bone marrow grafting at various dose levels (10 Gy, 2 Gy, 1.35 Gy); 10 patients received a localized irradiation of 2 Gy in the Waldeyer ring and the remainder 13 patients had a localized pancreatic irradiation. Hyperamylasemia was found to be constant and dose-dependent in TBI and Waldeyer ring patients. In contrast, patients given a pancreatic irradiation did not show any increase in amylasemia. Dubray et al. [20] measured serum salivary amylase before and 24 h after either TBI (31 patients) or localized irradiation including the salivary glands (40 patients) or the pancreatic area (22 patients). They proposed, on the basis of a simple mathematical model of the increase in amylasemia, that irradiation of salivary glands predicted a maximum amylasemia level for doses larger than 4 Gy and smaller than 10 Gy, given in a fractionated setting. They stressed that post-RT hyperamylasemia is a good criterion for triage of accidentally irradiated patients.

### Consideration

The usual total RT dose given to HNC ranges between 60–70 Gy (2 Gy fraction) and it depends on stage disease at diagnosis and subsequent multimodality treatment approach. Last decades are characterized by improvement in RT technology and this guarantees higher rates of salivary glands sparing. In RT of the head and neck region, the parotid gland tissue is one of the most critical dose-limiting organs. Well-known side effects, such as xerostomia, dental caries and osteoradionecrosis, are routinely observed, whereas the effect of salivary gland tissue irradiation on serum amylase levels received relatively little attention. RT induces an acute increase in salivary amylase in serum and patient can show clinical evidence of parotitis consistent with it. For instance, based on PARSPORT trial data, intensity modulated RT (IMRT), compared with conventional 3-dimensional RT, significantly reduced the incidence of xerostomia and resulted in better recovery of saliva secretions [21]. But detailed analysis of amylase quantitative measurements was not recorded. The reduction in parotid cell production of saliva may be related with decreased production of amylase enzyme as well. A relationship between acute increase and chronic decrease in serum salivary amylase and subsequent xerostomia is logical but has not been established. This event could be associated to late toxicity, which makes a great impact on clinical evaluation and results from a different radiation targets within the tissue. Further studies are needed to verify if any long term changes in salivary amylase levels occur. Probably serum salivary amylase could be used as a biological indicator. Its dose-effect relationship needs to be more precisely defined, especially because of a considerable inter-individual variability. Measurement of amylase activity may be used as an end point for radiation damage in HNC patients to identify patients at increased or decreased risk for RT-related injury. An indicator of radiation tolerance could be increasingly important due to the growing number of HNC treatment sur*vivo*rs. It would maximize individual therapeutic gain.

## CONCLUSIONS

Serum salivary amylase biochemical changes after irradiation in HNC patients are less commonly appreciated in clinical trials. At present, no consistent and validated predictive assay exists that is able to definitively associate salivary amylase alteration to salivary gland damage. Actually, the information obtained from the published studies is limited and most of the available references are old, which might mean the topic is not frontier research. But we believe that serum salivary amylase activity is valuable to a certain extent to clinical measure. An improved understanding of the radiobiological mechanism, as well as appropriate clinical data collection, could represent the key to construct appropriate dose-effect curves to assess the degree of radiation-induced damage in HNC clinical practice.
